# Patterns of surveillance for late effects of BCR-ABL tyrosine kinase inhibitors in survivors of pediatric Philadelphia chromosome positive leukemias

**DOI:** 10.1186/s12885-021-08182-z

**Published:** 2021-04-29

**Authors:** Stephanie M. Smith, Himalee S. Sabnis, Rebecca Williamson Lewis, Karen E. Effinger, John Bergsagel, Briana Patterson, Ann Mertens, Kathleen M. Sakamoto, Lidia Schapira, Sharon M. Castellino

**Affiliations:** 1 Division of Hematology/Oncology, Department of Pediatrics, Stanford University School of Medicine, Stanford, California USA; 2 Department of Pediatrics, Division of Hematology/Oncology/Bone Marrow Transplantation, Emory University, Atlanta, GA USA; 3 Aflac Cancer and Blood Disorders Center, Children’s Healthcare of Atlanta, Atlanta, GA USA; 4 Department of Pediatrics, Division of Endocrinology, Emory University, Atlanta, GA USA; 5 Stanford Cancer Institute and Division of Medical Oncology, Department of Medicine, Stanford University School of Medicine, Stanford, California USA

**Keywords:** Tyrosine kinase inhibitors, Bcr-abl leukemia, Late-effects, Surveillance, CML, Ph + ALL

## Abstract

**Background:**

Targeted anticancer therapies such as BCR-ABL tyrosine kinase inhibitors (TKIs) have improved outcomes for chronic myeloid leukemia (CML) and Philadelphia chromosome-positive acute lymphoblastic leukemia (Ph + ALL). However, little is known about long-term risks of TKIs in children. Exposure-based survivorship guidelines do not include TKIs, thus surveillance practices may be variable.

**Methods:**

We retrospectively examined surveillance for cardiac and endocrine late effects in children receiving TKIs for Ph + leukemias, diagnosed at < 21 years between 2000 and 2018. Frequency of echocardiogram (ECHO), electrocardiogram (EKG), thyroid stimulating hormone (TSH), dual-energy x-ray absorptiometry (DXA), and bone age testing were abstracted. Descriptive statistics were stratified by leukemia type.

**Results:**

66 patients (CML *n* = 44; Ph + ALL *n* = 22) met inclusion criteria. Among patients with CML, ≥1 evaluation was done: ECHO (50.0%), EKG (48.8%), TSH (43.9%), DXA (2.6%), bone age (7.4%). Among patients with Ph + ALL, ≥1 evaluation was done: ECHO (86.4%), EKG (68.2%), TSH (59.1%), DXA (63.6%), bone age (44.4%). Over a median 6.3 and 5.7 years of observation, respectively, 2% of patients with CML and 57% with Ph + ALL attended a survivorship clinic.

**Conclusions:**

Despite common exposure to TKIs in survivors of Ph + leukemias, patterns of surveillance for late effects differed in CML and Ph + ALL, with the latter receiving more surveillance likely due to concomitant chemotherapy exposures.

Targeted therapies such as TKIs are revolutionizing cancer treatment, but surveillance for late effects and referral to survivorship clinics are variable despite the chronicity of exposure. Evidence based guidelines and longer follow-up are needed.

**Supplementary Information:**

The online version contains supplementary material available at 10.1186/s12885-021-08182-z.

## Background

Outcomes for pediatric malignancies associated with the Philadelphia chromosome have improved since the introduction of BCR-ABL tyrosine kinase inhibitors (TKIs) in the early 2000s. In chronic myeloid leukemia (CML), long-term treatment with a single agent TKI such as imatinib, dasatinib, or nilotinib has been efficacious in inducing durable cytogenetic remissions [1–5]. In Philadelphia chromosome-positive acute lymphoblastic leukemia (Ph + ALL), survival rates have improved with the addition of imatinib or dasatinib to intensive multiagent chemotherapy [6, 7]. Notably, TKIs have dramatically reduced the need for hematopoietic stem cell transplantation (HSCT) in both CML and Ph + ALL [6–8].

While there are clear benefits associated with TKI use in CML and Ph + ALL, it remains important to consider the risk of off-target effects in young patients exposed to these agents over many years [9, 10]. Alteration of bone metabolism has been well described following prolonged exposure to imatinib and dasatinib for CML [11, 12]. We and others have demonstrated diminished linear growth during long-term treatment with these agents [13–17]. While the incidence of cardiovascular disease such as myocardial infarction, peripheral arterial disease and stroke is elevated in adults with CML treated with second and third generation TKIs, no studies have systematically examined these effects in children [18, 19]. In Ph + ALL, the risk of late effects from combining TKIs and chemotherapy may be increased compared to that of chemotherapy or TKIs alone. Although some pediatric clinical trials for both CML (NCT00777036, NCT01844765) and Ph + ALL (NCT00720109, NCT01460160, NCT03007147) required periodic bone health, thyroid, and cardiac surveillance to evaluate for potential long-term toxicities of TKIs, results are not yet available.

The Children’s Oncology Group (COG) has published exposure-based guidelines for screening for late effects of cancer treatment in children and adolescents [20, 21]. To date neither the COG nor the National Comprehensive Cancer Network (NCCN) guidelines include recommendations for screening for late effects emerging after targeted therapies such as TKIs [22]. We evaluated real-world surveillance practices for potential cardiac and endocrine late effects of TKIs in pediatric and adolescent/young adult (AYA) survivors of CML and Ph + ALL.

## Methods

We identified a cohort of children and AYAs who were diagnosed with CML or Ph + ALL at < 21 years, received TKI therapy between January 1, 2000 and July 31, 2018, and had ≥1 year of follow-up at two large cancer centers – Aflac Cancer and Blood Disorders Center at Children’s Healthcare of Atlanta (CHOA) and Stanford University Medical Center (including Lucile Packard Children’s Hospital at Stanford and Stanford Health Care). Patients were identified by institutional cancer registries and confirmed by querying electronic medical records (EMR) at each institution. Patients with CML were eligible if they were treated with a TKI and ≥ 1 year from diagnosis. Patients with Ph + ALL were eligible after completion of frontline chemotherapy regimens containing a TKI. Patient demographics, diagnosis, treatment details, occurrence of secondary neoplasm and vital status were abstracted. Data were censored at entry into a prospective TKI screening study at CHOA, hematopoietic stem cell transplantation (HSCT), subsequent malignant neoplasm (SMN), relapse (Ph + ALL), transformation to blast crisis (CML), death, transition of care, loss to follow-up, or on July 31, 2019, whichever occurred first.

Primary outcomes included frequency of surveillance for cardiac and endocrine health. Investigations done within a month from diagnosis were considered baseline studies and were excluded from the analysis. Surveillance echocardiograms (ECHO), electrocardiograms (EKG), thyroid function tests (thyroid stimulating hormone [TSH]), dual-energy x-ray absorptiometry (DXA), or bone-age identified in the institutional EMRs during the observation period were included. Inclusion of DXA was limited to patients ≥5 years of age at diagnosis, and bone age to boys < 16 years and girls < 15 years at diagnosis. Evaluation of surveillance tests for patients with Ph + ALL was limited to the time period after completion of upfront chemotherapy regimens and excluded tests done after a relapse in order to focus on late effect surveillance rather than acute treatment needs. Tests were categorized according to whether they were mandated by a clinical trial at protocol-specified time points for enrolled patients (Supplemental Table 1 and 2).

Data were collected using Research Electronic Data Capture (REDCap) tools hosted at CHOA and Stanford [23]. De-identified datasets were combined for analysis at CHOA. The study was approved by Emory University, CHOA, and Stanford University Institutional Review Boards. Descriptive statistics were computed, including number and percentage for categorial variables, and median and range for continuous variables. Results were stratified by diagnosis. The proportion of patients who underwent each surveillance test accounting for clinical trial requirements was determined. Among those who had more than one of the surveillance tests, the median time between the tests was calculated. Analyses were performed in SAS version 9.3 (Cary, NC) and figures were designed in Microsoft Excel.

## Results

We identified 66 patients who received TKI for CML (*n* = 44) or Ph + ALL (*n* = 22). Overall, 27% of patients were non-Hispanic white, 23% were non-Hispanic black, 20% were Hispanic, and 24% were Asian.

### CML

Among 44 patients with CML (Table 1), the median age at diagnosis was 13 years. The median duration of TKI treatment was 6.3 years (range: 0.9, 15.6), and median follow-up was 6.3 years (range: 1.1, 15.8). Patients most commonly received imatinib (79.5%), and 47.7% changed TKIs during the observation period with dasatinib being the second most common exposure (59.1%). Six patients (13.6%) were enrolled on a clinical trial for CML.

**Table 1:** Characteristics of Ph + leukemia survivors by diagnosis

	CML (***N*** = 44)	Ph + ALL (***N*** = 22)
	N (%)	N (%)
**Clinical Characteristics**		
Sex		
Male	21 (47.7)	12 (54.5)
Female	23 (52.3)	10 (45.5)
Race and ethnicity		
White non-Hispanic	11 (25.0)	7 (31.8)
Black non-Hispanic	12 (27.3)	3 (13.6)
Hispanic	5 (11.4)	8 (36.4)
Asian	14 (31.8)	2 (9.1)
Other/Not Stated	2 (4.5)	2 (9.1)
Age at diagnosis, years	13 (3, 20)*	11 (3, 20)*
Age at censoring, years	19 (6, 35)*	17.5 (6, 26)*
Time under observation, years	6.3 (1.1, 15.8)*	5.7 (2.1, 11.8)*
**Treatment Information**		
Tyrosine kinase inhibitor		
Imatinib	35 (79.5)	14 (63.6)
Dasatinib	26 (59.1)	16 (72.7)
Nilotinib	7 (15.9)	0 (0.0)
Ponatinib	2 (4.5)	0 (0.0)
Bosutinib	1 (2.3)	0 (0.0)
More than one TKI	21 (47.7)	8 (36.4)
Duration of TKI treatment, years^a^	6.3 (0.9, 15.6)*	2.8 (0.6, 11.6)*
Cranial radiation		
Yes	0 (0.0)	6 (27.3)
No	44 (100.0)	16 (72.7)
Clinical trial enrollment		
Enrolled	6 (13.6)	9 (40.9)
Not enrolled	38 (86.4)	13 (59.1)

* Median (minimum, maximum) for continuous variables

^a^TKI treatment duration for Ph + ALL reflects 1 patient who completed all planned therapy in 0.6 years (hyper-CVAD + TKI) and 6 patients who continued single-agent TKI after upfront therapy

**Fig. 1:**
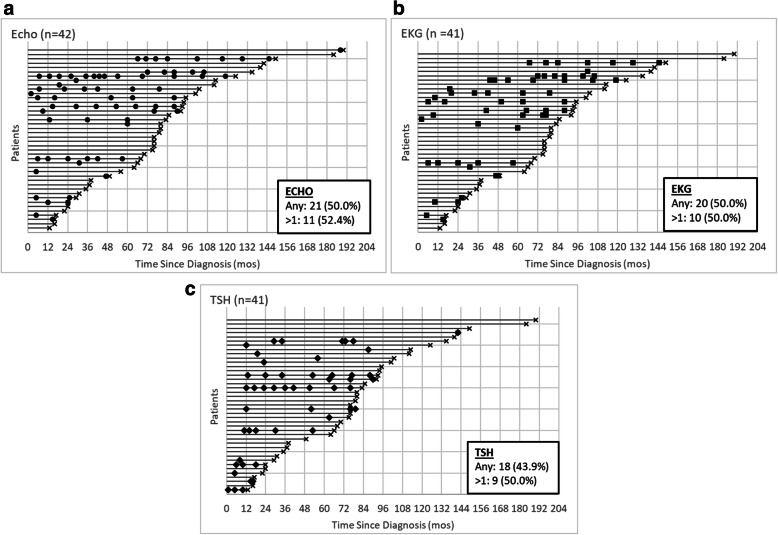
Cardiac and endocrine surveillance independent of clinical trial requirements in patients with CML. **a** Echocardiogram (ECHO) – each circle is an ECHO. **b** Electrocardiogram (EKG) – each square is an EKG. **c** Thyroid stimulating hormone (TSH) – each diamond is a TSH. Each line represents a patient. Lines begin at diagnosis (time 0) and continue until censoring (black X). Solid-filled shapes identify surveillance done outside of clinical trial requirements. Patients with clinical trial requirements for surveillance (*N =* 2 ECHO, *N* = 3 EKG, TSH) are not included in the charts. DXA is not shown because only one patient had a DXA outside of clinical trial requirements

Patterns of cardiac and endocrine surveillance during the observation period are illustrated in Fig. 1. A minority of patients had clinical trial requirements for surveillance and are not shown in the figure. Two patients had annual ECHOs as a clinical trial requirement. Among the other 42 patients, 21 (50%) had an ECHO, of whom 11 (52.4%) had more than one. Three patients were enrolled on trials with annual EKG, TSH, DXA, and bone age requirements. Of the remaining 41 patients, 20 (48.8%) had an EKG, of whom 10 (50%) had more than one. TSH was checked in 18 patients (43.9%) and 9 (50%) had more than one. Only one patient had a DXA and two patients had bone age assessments outside of the clinical trial setting. Considering the 39 patients who were not enrolled on a trial with ECHO, EKG, or TSH requirements, 13 (33%) had none of the surveillance tests, 12 (31%) had all three tests, and 14 (36%) had one or two of the three tests. One patient with CML (2%) attended a survivorship clinic annually during the observation period, beginning 5.3 years after diagnosis.

### Ph + ALL

Among 22 patients with Ph + ALL (Table 1), the median age at diagnosis was 11 years. Most patients received dasatinib (72.7%) or imatinib (63.6%) during their treatment; eight (36.4%) changed TKIs at least once during the observation period. Nine (40.9%) were enrolled on a clinical trial for Ph + ALL treatment. Frontline chemotherapy and TKI regimens were a median of 2.7 years in duration (range: 0.6, 3.4). Six patients (27.3%) continued single agent TKI after completion of upfront therapy. Patients were followed for a median of 5.7 years (range: 2.1, 11.8) before censoring. One patient developed monosomy 7 associated myelodysplastic syndrome 3.3 years after diagnosis.

**Fig. 2:**
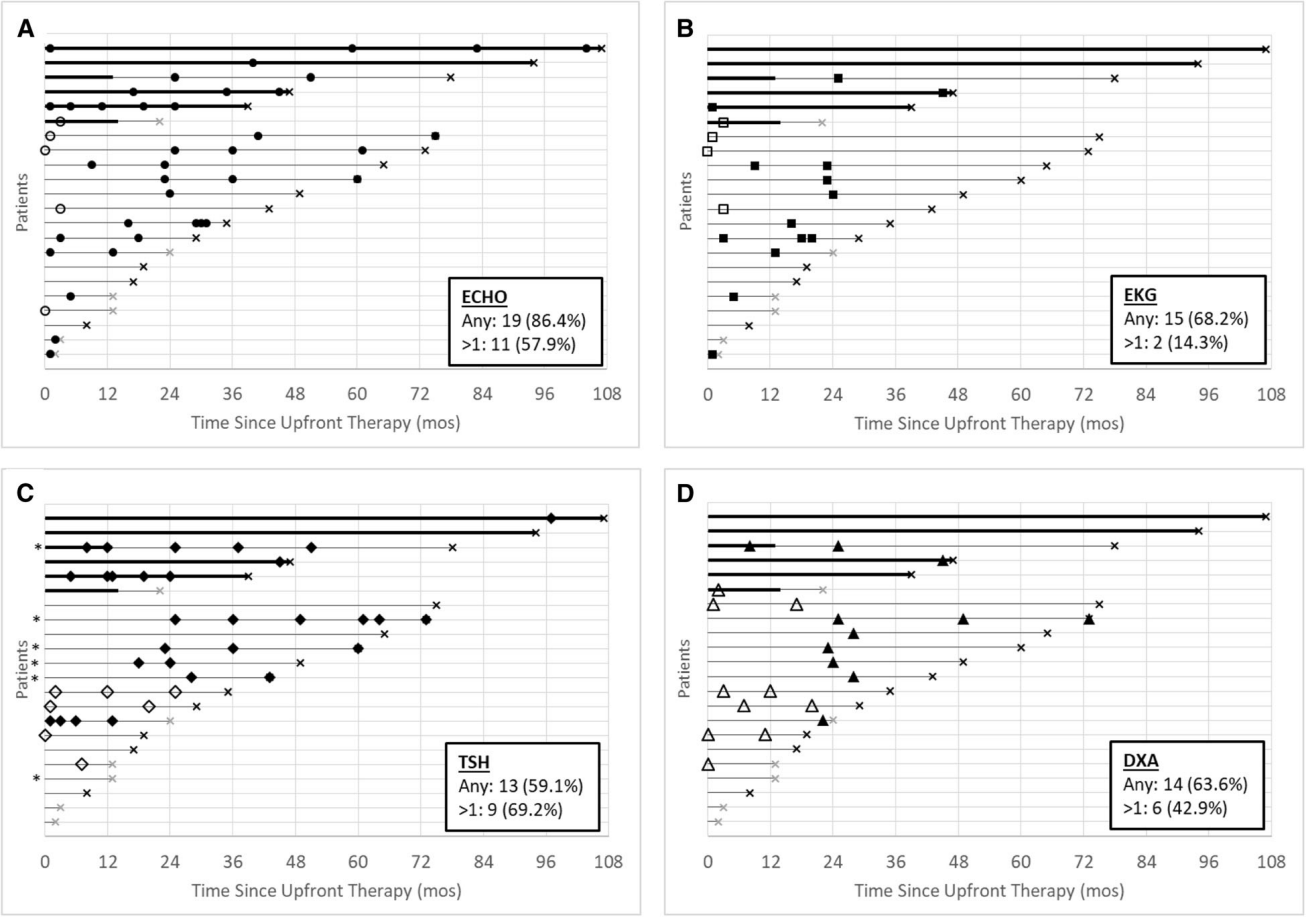
Cardiac and endocrine surveillance after completion of upfront therapy in patients with Ph + ALL. **a** Echocardiogram (ECHO) – each circle is an ECHO. **b** Electrocardiogram (EKG) – each square is an EKG. **c** Thyroid stimulating hormone (TSH) – each diamond is a TSH. **d** Dual-energy x-ray absorptiometry (DXA) – each triangle is a DXA. Each line represents a patient. Lines begin at completion of upfront therapy (time 0) and continue until censoring (black X) or relapse (grey X). Unfilled shapes identify surveillance done as a clinical trial requirement. Solid-filled shapes identify surveillance done outside of clinical trial requirements. Bolded timeline indicates single-agent TKI therapy after upfront therapy. For TSH, an asterisk indicates the patient received cranial radiation

The individual course of cardiac and endocrine surveillance after completion of upfront therapy is illustrated in Fig. 2 for each patient in their follow-up period. During the observation period, 19 of 22 (86.4%) patients had at least one ECHO, of whom 11 (57.9%) had more than one. The three patients with no off-therapy ECHOs had less than 20 months of follow-up and had no clinical trial requirements for off-therapy ECHOs. Fifteen (68.2%) patients had at least one EKG, of whom two (13.3%) had more than one. Among the seven patients with no EKGs, five had less than 24 months of follow-up and two continued on long-term TKI monotherapy**.** Thirteen (59.1%) patients had TSH levels checked; among them, five had received cranial radiation, four had a clinical trial requirement for TSH levels, and three were on long-term TKI monotherapy. Overall, 69.2% (9/13) had more than one TSH. DXA scans were completed in 14 (63.6%) patients, six of whom had a clinical trial requirement. For the eight patients without a clinical trial requirement, receipt of a DXA tended to cluster around 24 months off-therapy. Among the six patients who continued on single agent TKI after completion of upfront therapy, the two with the longest follow-up had no EKGs or DXAs over more than 7 years of observation. Bone age assessments were completed in 8/18 (44.4%); six had a clinical trial requirement and two did not. Considering all off-therapy surveillance tests, a minority of ECHOs, EKGs, and TSHs were obtained as clinical trial requirements (5/42 ECHOs, 4/18 EKGs, 7/36 TSHs). In contrast, almost half of the DXAs (10/21) and nearly all bone age assessments (11/13) were required by a clinical trial. Among the 14 patients who were at least 2 years from completion of upfront therapy, eight (57.1%) attended a survivorship clinic, one of whom was still receiving TKI at the time of the survivorship visit.

## Discussion

With the increasing application and efficacy of TKIs in Ph + malignancies, there is a growing need to understand the risk of potential long-term off target effects [10, 24, 25]. Little is known about absolute risk or about how pediatric and adolescent patients are monitored for potential toxicities after novel therapies [20]. We present the first real-world description of surveillance practices in an ethnically and racially diverse cohort of Ph + leukemia survivors receiving TKIs over a 15-year period, accounting for clinical trial requirements that impact surveillance practice.

Our study highlights the differences in patterns of surveillance for late effects when TKI is used as monotherapy in CML compared to when it is combined with conventional chemotherapy in Ph + ALL. Overall, we found that no more than half the patients on chronic TKIs for CML underwent cardiac or endocrine surveillance at any time during their treatment. In contrast, patients with Ph + ALL and TKI exposure of shorter duration tended to have more cardiac and endocrine surveillance. This is attributed to the fact that Ph + ALL patients receive TKIs in combination with conventional chemotherapies for which COG LTFU exposure-based guidelines recommend specific surveillance (i.e., ECHO and EKG due to anthracycline chemotherapy, annual TSH in those who received cranial radiation, and DXA upon entry into long-term follow-up care due to corticosteroids and methotrexate) [26]. In our cohort, many Ph + ALL survivors had testing at a frequency that was not entirely explained by COG guidelines for conventional therapy or by clinical trial requirements. This pattern of testing after entry into survivorship may be attributed to individual patient clinical course or comorbidities (i.e., more frequent cardiac surveillance due to an underlying condition). Whether any of the surveillance was due to the TKI exposure itself (i.e., provider concern about risks of TKIs) could not be assessed in our retrospective study.

Surveillance for late effects in asymptomatic patients after pediatric cancer treatment is largely directed by childhood cancer survivorship guidelines [20, 26, 27]. However, the development of evidence-based guidelines for late effects surveillance after TKIs has been limited by small patient numbers, the relatively recent integration of these agents into pediatric oncology practice, and the lack of biomarkers for off-target injury. Currently no guidelines exist for surveillance of *asymptomatic* patients for long-term or late effects of TKIs aside from the NCCN guidelines recommending regular EKGs in CML patients taking nilotinib due to risk of QTc-prolongation [22]. For *symptomatic* patients, the NCCN guidelines for CML comment on the utility of DXA and bone age [22]. The COG LTFU guidelines do not currently include guidance for *asymptomatic* or *symptomatic* patients exposed to targeted therapies [20]. Although pediatric CML experts have embedded recommendations for cardiac and endocrine surveillance within CML publications, the type and frequency of testing for asymptomatic patients is not consistent across publications [24, 28–30]. For example, recommendations for TSH screening vary from none to yearly, DXA from none to every 5 years to yearly, and ECHO and EKG from none to yearly, depending on the publication.

Consistent with the lack of evidence-based guidelines, we found that surveillance practices were highly variable among patients with CML. The pattern of receipt of certain surveillance tests (ECHO, EKG, TSH) appeared to track together, with one-third having none of these tests. Few patients with CML had a bone age or DXA during TKI treatment in the absence of a clinical trial requirement, limiting an understanding of the true incidence and temporal pattern of subclinical growth and/or bone health abnormalities. An increasing body of literature now indicates an association between long-term TKI therapy in early childhood and adverse effects on linear growth [13, 14, 16, 17, 31]. Preclinical models and case series suggest that TKIs may delay bone age and increase the risk of low bone mineral density [15, 32]. However, the optimal surveillance schedule has not been determined. The lack of national evidence-based guidelines for late effects of TKIs and uncertain risk of cardiac and endocrine late effects in children and adolescents are the most likely explanation for the low rates of surveillance in our cohort.

TKI monotherapy of long and undefined duration, as used for CML, represents a new paradigm in pediatric oncology. Most pediatric patients are currently prescribed TKIs indefinitely. During this time, they remain in the care of the primary oncologist with a focus on active cancer treatment and molecular monitoring and are therefore less likely to transition to survivorship care [24]. Notably, only one patient with CML in our cohort was seen in a survivorship clinic, despite institutional commitment to survivorship care across both centers [33–35]. Survivorship clinics are traditionally reserved for patients who have completed all cancer-directed therapy, as surveillance for late effects is the focus. Our data indicate high rates of transition to survivorship for Ph + ALL survivors, yet underscore the need for further study to determine the ideal model for shared care addressing survivorship issues for patients receiving chronic cancer-directed therapy, such as those with CML [36].

Turning to Ph + ALL, recent pediatric clinical trials have included secondary or exploratory aims to evaluate long-term toxicities of TKIs in these patients who are also treated with intensive chemotherapy. For example, two studies (NCT00720109, NCT01460160) required yearly TSH, DXA, and bone age for the first 5 years after completion of therapy, while another (NCT00022737) required an ECHO and EKG upon completion of therapy and 1 year later (Supplemental Table 2). The ongoing COG/EsPhALL study (NCT03007147) requires an ECHO and bone age upon completion of therapy and 3 years later but does not require TSH levels or DXA scans. Although the yield of this more frequent surveillance is not yet known, the hope is that these studies will soon provide evidence of the pattern and course of subclinical toxicities. Since most clinical trials end routine surveillance 5 years from completion of therapy, the need remains for longer follow-up to detect off-target effects of TKIs as monotherapy or in combination with known cardiotoxins or endocrine disruptors.

Our retrospective study focused on the clinical experience at two institutions, which may limit the generalizability of our results. At the same time, the heterogeneity in treatment exposures and settings within the two institutions yields real world experience given the lack of evidence-based guidelines and the gap in our understanding of when patients on new agents should be seen in survivorship clinics. Among CML patients age 18–20 years, some were treated at the pediatric center (*N* = 2) or adult center (*N* = 7) at one institution, while all were treated at the pediatric center at the other institution; this echoes the heterogeneity in AYA care nationally. In this study, we considered all BCR-ABL TKIs together when examining surveillance because the sample size limited our ability to reliably evaluate surveillance patterns among subgroups of patients treated with different TKIs, given the crossover of agents for many patients. Finally, our retrospective study design limited our analysis to data that were available in the EMR. As a result, we do not know the reason that a test was done (i.e. symptom-triggered, surveillance due to TKI risk or chemotherapy risk, or for another reason). However, for new agents with unknown long-term toxicity profiles (such as BCR-ABL TKIs), there is still value in examining the frequency, and in the future, examining the yield of surveillance tests. Given the lack of uniformity in surveillance practices, we did not evaluate the yield of surveillance for abnormalities, as this type of analysis is most accurate when surveillance occurs at standardized time points.

## Conclusions

In summary, the variability of surveillance practices identified in this study calls attention to the importance of standardizing surveillance guidelines for cancer survivors exposed to new agents such as TKIs (with and without traditional chemotherapy) and the need for biomarkers of subclinical injury from TKIs. Our data also highlight the opportunity to explore shared management of patients on chronic anticancer therapy with survivorship teams. Pediatric oncologists often consider the risk of late effects when selecting among upfront therapy options, but we need more data to do so effectively for patients with CML and Ph + ALL, for whom multiple BCR-ABL TKIs may be considered. With the rapidly expanding use of novel therapies, concerted efforts are needed to identify emerging long-term and late toxicities and prospective surveillance studies may be one step toward this goal [10, 25, 33].

## Supplementary Information

**Additional file 1: Supplemental Table 1.** Cardiac and endocrine surveillance requirements on select pediatric CML clinical trials. **Supplemental Table 2.** Cardiac and endocrine surveillance requirements after completion of upfront therapy on select pediatric Ph + ALL clinical trials.

## Data Availability

The datasets analyzed during the study are not publicly available due to patient privacy and institutional data sharing regulations.

## Abbreviations

CML: Chronic Myeloid Leukemia
Ph + ALL: Philadelphia Chromosome-positive Acute Lymphoblastic Leukemia
TKI: Tyrosine Kinase Inhibitor
COG: Children’s Oncology Group
AYA: Adolescent and Young Adults
DXA: Bone Density Scan
