# Supplementary material for: Patterns of surveillance for late effects of BCR-ABL tyrosine kinase inhibitors in survivors of pediatric Philadelphia chromosome positive leukemias
Source: BMC Cancer. 2021 Apr 29;21:474. doi: 10.1186/s12885-021-08182-z (PMC8082962; doi:10.1186/s12885-021-08182-z)
Supplement: Supplementary file 1 — Additional file 1: Supplemental Table 1. Cardiac and endocrine surveillance requirements on select pediatric CML clinical trials. Supplemental Table 2. Cardiac and endocrine surveillance requirements after completion of upfront therapy on select pediatric Ph + ALL clinical trials. [file 12885_2021_8182_MOESM1_ESM.docx]

**Supplemental Table 1.** Cardiac and endocrine surveillance requirements on select pediatric CML clinical trials

|  | **NCT00004932**  **POG-9973 (phase I imatinib)** | **NCT00030394**  **AAML0123 (phase II imatinib)** | **NCT00777036**  **CA180-226 (phase II dasatinib)** | **NCT01844765**  **AAML1321 (phase II nilotinib)** |
| --- | --- | --- | --- | --- |
| Number enrolled | N=1 | N=2 | N=2 | N=1 |
| **ECHO** |  |  |  |  |
| Year 0 (1-11 mos) |  | X |  |  |
| Year 1 (12-23 mos) |  | X | X |  |
| Year 2 (24-35 mos) |  |  | X |  |
| Year 3 (36- 47 mos) |  |  | X |  |
| Year 4 (48-59 mos) |  |  | X |  |
| Year ≥ 5 (60+ mos) |  |  | X |  |
| **EKG** |  |  |  |  |
| Year 0 (1-11 mos) |  |  | X | X |
| Year 1 (12-23 mos) |  |  | X | X |
| Year 2 (24-35 mos) |  |  | X | X |
| Year 3 (36- 47 mos) |  |  | X | X |
| Year 4 (48-59 mos) |  |  | X | X |
| Year ≥ 5 (60+ mos) |  |  | X | X |
| **TSH** |  |  |  |  |
| Year 0 (1-11 mos) |  |  |  |  |
| Year 1 (12-23 mos) |  |  | X | X |
| Year 2 (24-35 mos) |  |  | X | X |
| Year 3 (36- 47 mos) |  |  | X | X |
| Year 4 (48-59 mos) |  |  | X | X |
| Year ≥ 5 (60+ mos) |  |  | X | X |
| **DXA*** |  |  |  |  |
| Year 0 (1-11 mos) |  |  |  |  |
| Year 1 (12-23 mos) |  |  | X | X |
| Year 2 (24-35 mos) |  |  | X | X |
| Year 3 (36- 47 mos) |  |  | X | X |
| Year 4 (48-59 mos) |  |  | X | X |
| Year ≥ 5 (60+ mos) |  |  | X | X |
| **Bone Age** |  |  |  |  |
| Year 0 (1-11 mos) |  |  |  |  |
| Year 1 (12-23 mos) |  |  | X | X |
| Year 2 (24-35 mos) |  |  | X | X |
| Year 3 (36- 47 mos) |  |  | X | X |
| Year 4 (48-59 mos) |  |  | X | X |
| Year ≥ 5 (60+ mos) |  |  | X | X |

Abbreviations: mos = months (time since start of therapy); ECHO = echocardiogram; EKG = electrocardiogram; TSH = thyroid stimulating hormone; DXA = dual-energy x-ray absorptiometry

*DXA only if age ≥ 5 years per protocol

**Supplemental Table 2.** Cardiac and endocrine surveillance requirements after completion of upfront therapy on select pediatric Ph+ ALL clinical trials

|  | **NCT00022737**  **AALL0031 (imatinib)** | **NCT00720109**  **AALL0622 (dasatinib)** | **NCT01460160**  **AALL1122 (dasatinib)** |
| --- | --- | --- | --- |
| Number enrolled | N=3 | N=2 | N=4 |
| **ECHO** |  |  |  |
| Off-therapy Yr 0 (1-11 mos) | X | X |  |
| Off-therapy Yr 1 (12-23 mos) | X | X |  |
| Off-therapy Yr 2 (24-35 mos) |  |  |  |
| Off-therapy Yr 3 (36- 47 mos) |  |  |  |
| Off-therapy Yr 4 (48-59 mos) |  |  |  |
| Off-therapy ≥ 5 Yr (60+ mos) |  |  |  |
| **EKG** |  |  |  |
| Off-therapy Yr 0 (1-11 mos) | X | X |  |
| Off-therapy Yr 1 (12-23 mos) | X | X |  |
| Off-therapy Yr 2 (24-35 mos) |  |  |  |
| Off-therapy Yr 3 (36- 47 mos) |  |  |  |
| Off-therapy Yr 4 (48-59 mos) |  |  |  |
| Off-therapy ≥ 5 Yr (60+ mos) |  |  |  |
| **TSH** |  |  |  |
| Off-therapy Yr 0 (1-11 mos) |  | X | X |
| Off-therapy Yr 1 (12-23 mos) |  | X | X |
| Off-therapy Yr 2 (24-35 mos) |  | X | X |
| Off-therapy Yr 3 (36- 47 mos) |  | X | X |
| Off-therapy Yr 4 (48-59 mos) |  | X | X |
| Off-therapy ≥ 5 Yr (60+ mos) |  | X | X |
| **DXA*** |  |  |  |
| Off-therapy Yr 0 (1-11 mos) |  | X | X |
| Off-therapy Yr 1 (12-23 mos) |  | X | X |
| Off-therapy Yr 2 (24-35 mos) |  | X | X |
| Off-therapy Yr 3 (36- 47 mos) |  | X | X |
| Off-therapy Yr 4 (48-59 mos) |  | X | X |
| Off-therapy ≥ 5 Yr (60+ mos) |  | X | X |
| **Bone Age** |  |  |  |
| Off-therapy Yr 0 (1-11 mos) |  | X | X |
| Off-therapy Yr 1 (12-23 mos) |  | X | X |
| Off-therapy Yr 2 (24-35 mos) |  | X | X |
| Off-therapy Yr 3 (36- 47 mos) |  | X | X |
| Off-therapy Yr 4 (48-59 mos) |  | X | X |
| Off-therapy ≥ 5 Yr (60+ mos) |  | X | X |

Abbreviations: yr = year; mos = months (time since completion of therapy); ECHO = echocardiogram; EKG = electrocardiogram; TSH = thyroid stimulating hormone; DXA = dual-energy x-ray absorptiometry

*DXA only if age ≥ 5 years per protocol
